# Supplementary material for: Glycan Analysis as Biomarkers for Testicular Cancer
Source: Diagnostics (Basel). 2019 Oct 22;9(4):156. doi: 10.3390/diagnostics9040156 (PMC6963830; doi:10.3390/diagnostics9040156)
Supplement: Supplementary file 1 [file diagnostics-09-00156-s001.zip › Supp info file.docx]

**SUPPORTING INFORMATION FILE**

**Glycan analysis as biomarkers for testicular cancer**

**Michal Hires^a^, Eduard Jane^a^, Michal Mego^b,c^, Michal Chovanec^b^, Peter Kasak^d^, Jan Tkac^a,^**^[[1]](#footnote-1)^

^a^ Institute of Chemistry, Slovak Academy of Sciences, Dubravska cesta 9, 845 38 Bratislava, Slovakia;

^b^ Translational Research Unit, Faculty of Medicine, Comenius University and National Cancer Institute, Bratislava, Slovakia;

^c^ 2^nd^ Department of Oncology, Faculty of Medicine, Comenius University and National Cancer Institute, Bratislava, Slovakia;

^d^ Center for Advanced Materials, Qatar University, Doha 2713, Qatar

**Performance of glycosylated hCG as a TC biomarker**

We did evaluation how 5 single parameters (hCG, hCG-H, hCGβ, hCG-H% and hCGβ%) provided by prof. Cole *(1)* or their combination can discriminate between testicular GCT (TGCT) and other trophoblastic cancers (choriocarcinoma pre-therapy = ChCp, choriocarcinoma during therapy = ChCd and OGCC = ovarian germ cell cancer). The results really suggest that in order to discriminate TGCT from ChCp or from ChCd at least a combination of two biomarkers is needed in order to achieve AUC=1 (i.e. hCG + hCG-H; hCG + hCG-H%; or hCG-H + hCG-H%) (**Table S1a**). The results also suggest that it is not possible to discriminate between TGCT and OGCC with AUC=1 and that in order to achieve the highest possible AUC=0.889, three biomarkers need to be combined (hCG + hCG-H + hCGβ%). Similar analysis was applied to discriminate TGCT from several non-trophoblastic cancers with AUC= 1 obtained in nearly all cases (see **Table S1b**).

The results provided in **Table S1** really confirm that analysis of one biomarker might be problematic for diagnostic purposes and that for really reliable and robust TC diagnostics, prognosis or recurrence monitoring it might be useful to include glycans as TC biomarkers or to combine glycan biomarkers together with determination of protein levels in a form of multiparametric assays.

**Experimental section**

Machine learning (ML) is powerful tools with many applications. One of them is an application for clinical diagnostics to provide faster diagnoses, improved prediction performance, and reduced medical costs by eliminating of unnecessary testing. Several ML methods found application in clinical research and testing. Logistic regression is a well-suited for use in diagnostic models. Logistic regression allows only binomial outcomes e.g. evaluation healthy *vs.* sick. Logistic regression is regarded as a standard method for this type of analysis *(9)*. Logistic regression is probabilistic and is thus more intuitive than its non-probabilistic counterparts. Therefore it is easy interpretable and very popular in a clinical community. Generalization of logistic regression applied to categorical responses that have more than two categories is multinomial logit model. In this model for nominal response, variables simultaneously use all pairs of categories by specifying the odds of outcome in one category instead of another. The order of listing the categories is irrelevant with the main difference between these models in a form of an activation function.

In our case of MLR softmax function is used. All analyses were performed using R software v.3.4.4 *(10)* using CARET (**C**lassification **A**nd **RE**gression **T**raining) *(11)* with nnet *(12)* and glm package. 10-fold crossvalidation was used and AUC values were rounded on third decimal place.

**References**

1. Cole LA. Hyperglycosylated hcg drives malignancy in cancer cases. J Mol Oncol Res 2017;1:53-63.

1. Corresponding author, E-mail: [Jan.Tkac@savba.sk](mailto:Jan.Tkac@savba.sk), Tel.: 00421 2 5941 0263. [↑](#footnote-ref-1)
