# Supplementary material for: Glycan Analysis as Biomarkers for Testicular Cancer
Source: Diagnostics (Basel). 2019 Oct 22;9(4):156. doi: 10.3390/diagnostics9040156 (PMC6963830; doi:10.3390/diagnostics9040156)
Supplement: Supplementary file 1 [file diagnostics-09-00156-s001.zip › Table S1a.pdf]

	trophoblastic cancers	TGCT vs ChCp AUC	TGCT vs ChCd AUC	TGCT vs OGCC AUC
1	hCG	1	0,894	0,667
2	hCG-H	1	0,909	0,667
3	hCG-H% **(line3 / line2)**	0,546	1	0,722
4	hCGβ	0,889	0,727	0,583
5	hCGβ% **(line4 / line2)**	0,593	0,636	0,556
6	hCG + hCG-H	1	1	0,778
7	hCG + hCG-H%	1	1	0,806
8	hCG + hCGβ	1	0,924	0,722
9	hCG + hCGβ%	1	0,924	0,722
10	hCG-H + hCG-H%	1	1	0,806
11	hCG-H + hCGβ	1	0,924	0,667
12	hCG-H + hCGβ%	1	0,924	0,722
13	hCG-H% + hCGβ	0,907	1	0,833
14	hCG-H% + hCGβ%	0,704	1	0,778
15	hCGβ + hCGβ%	1	0,894	0,611
16	hCG + hCG-H + hCG-H%	1	1	0,833
17	hCG + hCG-H + hCGβ	1	1	0,861
18	hCG + hCG-H + hCGβ%	1	1	0,889
19	hCG + hCG-H% + hCGβ	1	1	0,833
20	hCG+ hCG-H % + hCGb %	1	1	0,833
21	hCG + hCGβ + hCGβ%	0,667	0,924	0,694
22	hCG-H + hCG-H% + hCGβ	1	1	0,833
23	hCG-H + hCG-H% + hCGβ%	1	1	0,833
24	hCG-H + hCGβ + hCGβ%	1	0,924	0,639
25	hCG-H% + hCGβ + hCGβ%	1	1	0,833
26	hCG + hCG-H+ hCG-H% + hCGβ	1	1	0,889
27	hCG + hCG-H + hCG-H% + hCGβ%	1	1	0,889
28	hCG + hCG-H + hCGβ + hCGβ%	1	1	0,861
29	hCG + hCG-H% + hCGβ + hCGβ%	1	1	0,861
30	hCG-H + hCG-H% + hCGβ + hCGβ%	1	1	0,861
31	hCG + hCG-H + hCG-H% + hCGβ + hCGβ%	1	1	0,861

hCG= hCG determination, hCG-H= hCG-H determination, hCGb= hCGb determination

ChCp = choriocarcinoma pre-therapy, ChCd = choriocarcinoma during therapy, OGCC = ovarian germ cell cancer, TGCT = testicular germ cell cancer,
BlC = bladder cancer, **BrC** = breast cancer, **CeC** = cervical cancer, **EnC** = endometrial cancer, **OvC** = ovarian cancer, **VuC** = vulvae cancer
