# Supplementary material for: Glycan Analysis as Biomarkers for Testicular Cancer
Source: Diagnostics (Basel). 2019 Oct 22;9(4):156. doi: 10.3390/diagnostics9040156 (PMC6963830; doi:10.3390/diagnostics9040156)
Supplement: Supplementary file 1 [file diagnostics-09-00156-s001.zip › Table S1b.pdf]

	Non-trophoblastic cancers	TGCT vs BlC AUC	TGCT vs BrC AUC	TGCT vs CeC AUC	TGCT vs EnC AUC	TGCT vs OvC AUC	TGCT vs VuC AUC
1	hCG	1	1	1	1	1	1
2	hCG-H	1	1	1	1	1	1
3	hCG-H% **(line3 / line2)**	0,4	0,667	0,7	0,667	0,833	0,667
4	hCGβ	1	1	1	1	1	1
5	hCGβ% **(line4 / line2)**	1	1	1	1	1	1
6	hCG + hCG-H	1	1	1	1	1	1
7	hCG + hCG-H%	1	1	1	1	1	1
8	hCG + hCGβ	1	1	1	1	1	1
9	hCG + hCGβ%	1	1	1	1	1	1
10	hCG-H + hCG-H%	1	1	1	1	1	1
11	hCG-H + hCGβ	1	1	1	1	1	1
12	hCG-H + hCGβ%	1	1	1	1	1	1
13	hCG-H% + hCGβ	1	1	1	1	1	1
14	hCG-H% + hCGβ%	1	1	1	1	1	1
15	hCGβ + hCGβ%	1	1	1	1	1	1
16	hCG + hCG-H + hCG-H%	1	1	1	1	1	1
17	hCG + hCG-H + hCGβ	1	1	1	1	1	1
18	hCG + hCG-H + hCGβ%	1	1	1	1	1	1
19	hCG + hCG-H% + hCGβ	1	1	1	1	1	1
20	hCG+ hCG-H % + hCGb %	1	1	1	1	1	1
21	hCG + hCGβ + hCGβ%	1	1	1	1	1	1
22	hCG-H + hCG-H% + hCGβ	1	1	1	1	1	1
23	hCG-H + hCG-H% + hCGβ%	1	1	1	1	1	1
24	hCG-H + hCGβ + hCGβ%	1	1	1	1	1	1
25	hCG-H% + hCGβ + hCGβ%	1	1	1	1	1	1
26	hCG + hCG-H+ hCG-H% + hCGβ	1	1	1	1	1	1
27	hCG + hCG-H + hCG-H% + hCGβ%	1	1	1	1	1	1
28	hCG + hCG-H + hCGβ + hCGβ%	1	1	1	1	1	1
29	hCG + hCG-H% + hCGβ + hCGβ%	1	1	1	1	1	1
30	hCG-H + hCG-H% + hCGβ + hCGβ%	1	1	1	1	1	1
31	hCG + hCG-H + hCG-H% + hCGβ + hCGβ%	1	1	1	1	1	1

hCG= hCG determination, hCG-H= hCG-H determination, hCGb= hCGb determination

ChCp = choriocarcinoma pre-therapy, ChCd = choriocarcinoma during therapy, OGCC = ovarian germ cell cancer, TGCT = testicular germ cell cancer,
BlC = bladder cancer, **BrC** = breast cancer, **CeC** = cervical cancer, **EnC** = endometrial cancer, **OvC** = ovarian cancer, **VuC** = vulvae cancer
